# The Effect of a Decade Implemented Project in Improving the Uptake of Comprehensive Contraception: Difference-In-Difference Analysis

**DOI:** 10.4314/ejhs.v33i6.2

**Published:** 2023-11

**Authors:** Addisalem Titiyos, Jemal Kassaw, Kathryn A O'Connell

**Affiliations:** 1 EngenderHealth Ethiopia country program office; 2 EngenderHealth HQ office

**Keywords:** ABRI, Contraceptive Effectiveness, difference-indifference analysis, Program Effectiveness, Program Evaluation

## Abstract

**Background:**

Promotion and use of family planning in countries with high birth rates have the potential to avert a third of all maternal deaths and nearly a tenth of childhood deaths. To support government efforts in creating wider access to comprehensive contraceptive methods, EngenderHealth has contributed to the government of Ethiopia's long-term goal of improving maternal health outcomes through its Access to Better Reproductive Health Initiative project

**Methods:**

Difference-in-Difference approach is the main methodology in this analysis to estimate the “contribution” or “effect” of the ABRI intervention by comparing the changes in family planning outcomes from 2005 to 2016 between the ABRI and non-ABRI areas. This analysis was based on pooled data from the 2005 and 2016 Ethiopian Demographic and Health Surveys. To track temporal changes in the family planning indicators in the ABRI and non-ABRI areas, we employed simple trend analysis.

**Results:**

The results show that overall contraceptive prevalence rate, use of injectables, women's knowledge of Long-Acting Reversible Contraception (LARC) methods, and their exposure to family planning information/messages from health workers all significantly improved in the ABRI intervention areas beyond what occurred in the non-ABRI areas. The greatest increase in the use of modern contraception was among adolescents aged 15-19 years, with a DID estimate of 22.4% (p=0.007), ABRI areas compared to no-ABRI areas.

**Conclusion:**

In the ABRI areas, family planning indicators recorded positive and significant changes. EngenderHealth has contributed its part in improving access to the uptake of comprehensive contraception and supporting government programs.

## Introduction

Promotion and use of family planning in countries with high birth rates have the potential to avert a third of all maternal deaths and nearly a tenth of childhood deaths (1). In the past 50 years, family-planning programs have played a major part in raising the prevalence of contraceptive use from less than 10% to 64% and reducing fertility in developing countries from six to about three births per woman (2). Ethiopia is among the countries with high fertility rates and the second-most populous country in Africa with an estimated population of over 113 million in 2019, and an annual population growth rate of 2.7% (3,4). According to EDHS 2016, about one in five currently married women in Ethiopia, has an unmet need for family planning, including 13% with an unmet need for spacing and 9% for limiting the number of children (5). The use of effective contraceptive methods has paramount importance to prevent the risk of unwanted pregnancies for women of reproductive age (6).

EngenderHealth contributed to the government of Ethiopia (GOE)'s long-term goal of improving maternal health outcomes through its Access to Better Reproductive Health Initiative (ABRI) project. The initiative aimed to support the government's efforts in creating wider access to comprehensive contraceptive methods. EngenderHealth implemented the ABRI project in six regional states and two city administrations between 2008 to 2019. The ABRI project supported the Ministry of Health's (MOH) health system and structures at all levels, with a focus on strengthening the capacity of woreda (district) health offices and health facilities, such as health centers and hospitals. Activities included training healthcare providers and managers, providing technical support such as supportive supervision, clinical monitoring and coaching, training competency assessment, and individual provider follow-up and support. The ABRI project also worked to link communities with the health system and health extension workers to deliver sustainable comprehensive contraception services.

Difference-In-Differences (DID) is a commonly utilized impact evaluation approach (7). This method compares before-after periods between treatment and control groups, providing an intuitive appeal that has been widely used in various fields, including health research. To the best knowledge of the authors, this is the first time this approach has been used for Ethiopia's DHS data. The objective of this study was to compare changes in key family planning indicators using the DID approach in ABRI areas compared to non-ABRI areas from 2005 to 2016, thereby contributing to the literature on evidence-based family planning interventions in Ethiopia.

## Materials and Methods

**Study design and setting**: This study employed a before-after design with control group method and used Difference-In-Difference (DID) analysis to explore and compare trends of family planning indicators in Ethiopia between 2005 and 2016, using pooled data from the 2005 and 2016 Ethiopian Demographic and Health Surveys (EDHS) (8). As the ABRI program intervention began in 2008, the 2005 EDHS served as a baseline. Due to the paucity of data, we used the 2016 EDHS as end-line, although the ABRI intervention was in operation until December 2019.

The EDHS clusters were reconstructed to create two broader domains, ABRI and non-ABRI areas, based on the woredas (districts) where ABRI operated until 2016. For this analysis, non-ABRI areas included the clusters and woredas that ABRI did not cover. The EDHS clusters are freshly sampled in every survey round and include over 600 clusters (Enumeration Areas). Details about the EDHS methodology, indicators, and questionnaires can be consulted elsewhere (8).

**Sample**: This analysis included women of reproductive age that were interviewed in the 2005 (n=12,201) and 2016 (n=13,861) EDHS. Of note, the ABRI area constituted 31.1% (n=3798) and 32.4% (n=4486) of the total sample in 2005 and 2016, respectively.

### Measurement

**Knowledge of LARC and permanent methods**: Women's awareness of LARC and permanent methods was assessed by asking the respondents if they had ever heard of family planning and, if so, they were asked to mention the type of family planning method they knew (prompted or unprompted). A woman was considered to have knowledge of LARC and permanent method if she mentioned spontaneously or promptly one or more of the following methods: IUD, Implants, female sterilization, and male sterilization. Here, we present three indicators (1) knowledge of one or more LARC, (2) knowledge of IUD, and (3) knowledge of Implants.

**Source information on family planning**: In the EDHS, women were asked about the different sources of family planning information they were exposed to in the 12 months before the interview. The possible sources included: (1) radio, (2) television, (3) home visit by a health worker or health extension worker that discussed family planning, and (4) in health facilities where health workers discussed family planning with the women.

**Current use of family planning**: The contraceptive prevalence rate (CPR) is defined as the percent of women of reproductive age (15-49 years) who are using (or whose partner is using) a contraceptive method at a particular point in time, almost always reported for women married or in sexual union (9). The modern contraceptive prevalence rate (mCPR) is measured based on those women in union who reported using modern methods. In this analysis, we focus on mCPR and current contraceptive use by type of method.

**mCPR trends by women's age**: In this section, we present trends in modern CPR from 2005 to 2016, stratified by age, in ABRI and non-ABRI areas. Three age categories are presented: 15-19, 20-24, and 25-49 years. Separate estimates for early adolescents aged 15-16 years were not possible due to small sample size, resulting in low precision for mCPR estimates. Separate urban and rural estimates, stratified by age, also suffer from small sample sizes and thus are not presented here.

**Informed choice**: As a proxy to family planning service quality, we examined the information given to current contraceptive users when given the current method, as reported by the women. These include information about side effects of the method, what to do if side effects are experienced, and information about other family planning methods.

**Data analysis**: To track temporal changes in the family planning indicators in the ABRI areas, we employed simple trend analysis. Difference-In-Difference (DID) approach is the main methodology in this analysis to estimate the “contribution” or “effect” of the ABRI intervention by comparing the changes in family planning outcomes from 2005 to 2016 between the ABRI and non-ABRI areas. In this study, we present unadjusted (crude) and adjusted DID estimates. We obtained adjusted DID estimates by running dummy-by-dummy interaction regression models to adjust for potential confounding factors, such as age, education, religion, and parity of the women.

In this analysis, we present data in three domains; i.e. rural, urban, and total (10). We used STATA version 14 (Stata Corporation, College Station, TX, USA) for data management and analyses. The survey command in STATA was used to declare the strata and primary sampling unit. Proportions, rates, coefficients, etc. were weighted for the sampling probabilities. A p-value less than 0.05 was considered statistically significant.

**Ethics**: Because this study used a secondary analysis of DHS data that is publicly available, no additional ethical approval was required.

## Results

**Characteristics of respondents**: There were no significant differences in women's age, education, marital status, religion, and parity between ABRI and non-ABRI areas in 2005 and 2016. The only statistically significant difference noted between ABRI and non-ABRI groups was the distribution of women by place of residence in 2016, where disproportionately more women lived in rural areas of non-ABRI (85.6%) than in the ABRI areas (73.2%). One-third of women's age group was 25-34 across comparison groups. Nearly two-thirds and half of the study participants were not able to read/write in 2005 and 2016, respectively, in both areas. Approximately 66% of the women were married and 30% had never gave birth to children across ABRI and non-ABRI areas (Table 1).

**Table 1:** Selected socio-demographic characteristics of women, ABRI vs. non-ABRI areas, 2005-2016

Socio-demographic characteristics	EDHS 2005		EDHS 2016	
	ABRI n=3798	Non-ABRI n=8403	ABRI n=4486	Non-ABRI n=9375
**Place of residence**				
Rural	86.3	87.0	73.2	85.6*
Urban (other than Addis Ababa)	13.7	13.0	26.8	14.3
**Age**				
15-19	23.5	22.9	20.3	21.8
20-24	17.4	17.9	18.3	17.2
25-34	30.2	31.2	34.2	33.7
35-49	28.9	28.0	27.2	27.3
**Education**				
Cannot read/write	67.8	68.9	45.0	51.9
Primary	22.9	21.8	37.4	34.1
Secondary +	9.3	9.3	17.6	14.0
**Marital Status**				
Never married	24.0	23.0	25.0	23.9
Married	66.2	66.5	65.2	67.4
Divorced/widowed	9.8	10.5	9.8	8.8
**Children ever born**				
0	30.4	29.1	31.7	30.7
1-2	18.4	20.3	23.6	20.7
3-4	17.7	18.0	18.3	18.2
5+	33.5	32.7	26.4	30.5
**Religion**				
Orthodox Christian	41.6	49.2	45.1	40.0
Muslim	34.9	28.1	26.1	34.2
Protestant	20.5	19.0	26.7	23.6
Others	3.0	3.7	2.1	2.2

* *p<0.05*

**Knowledge of one or more LARC and permanent methods**: In 2005, women in the ABRI and non-ABRI areas had comparable levels of knowledge at 48.3% and 49.4%, respectively. In 2016, the proportion of women in ABRI areas that reported knowing one or more LARC and permanent methods was 79% against 68.8% for women in non-ABRI areas. The DID estimate for this indicator was 9.1%, suggesting that women's knowledge of LARC and permanent methods in ABRI intervention areas increased on average 9.1% on the absolute scale more (p<0.001). The DID estimate in the rural areas was 7.1% (p=0.001), and this significant difference persisted after adjusting for women's age, education, religion, and parity with an adjusted DID of 6.8% (p<0.001).

As early as 2005, unlike other LARC and permanent methods, a much higher proportion of women in both areas reported knowing Implants (43.5% in the ABRI and 43.3% in the non-ABRI). In 2016, these proportions increased to 72.3% in ABRI areas and 61.9% in non-ABRI areas. The unadjusted DID associated with knowledge of Implants is estimated at 10.2% (p<0.001), and the adjusted is 7.3% (p<0.001) (Table 2). From 2005 to 2016, the proportion of rural women with knowledge of Implants increased from 37.4% to 65.8% in ABRI areas and from 37.7% to 58% in non-ABRI areas, with an adjusted DID estimate of 7.9% (p<0.001) (Table 2).

**Table 2:** Adjusted and unadjusted Difference-in-Difference (DID) estimates for Knowledge of one or more LARC & permanent method, IUD, and Implants, for all samples (total), rural and urban

	*Diff 2005= [ABRI 2005 - non-ABRI 2005]* *Diff 2016= [ ABRI 2016 - nonABRI 2016]* *DID=Dif 2016- Diff 2005*	Total		Rural		Urban	

Indicators		Estimate (%)	p-value	Estimate (%)	p-value	Estimate (%)	p-value
Knowledge of one or more LARC & permanent method	Unadjusted DID	9.1	<0.001	7.1	0.001	4.5	0.133
	Adjusted DID ^+^	6.5	<0.001	6.8	0.001	3.5	0.212
Knowledge of IUD	Unadjusted DID	4.8	0.008	3.4	0.082	3.1	0.498
	Adjusted DID^+^	2.8	0.102	2.9	0.132	2.4	0.589
Knowledge of Implants	Unadjusted DID	10.2	<0.001	8.1	<0.001	2.9	0.391
	Adjusted DID ^+^	7.3	<0.001	7.9	<0.001	1.7	0.579

+ DID with covariates, adjusted for women's age, education, religion, and parity.

**Source of information on family planning**: From 2005 to 2016, the proportion of women that reported listening to family planning messages on the radio in the previous 12 months declined both in ABRI (from 27.1% to 26%) and non-ABRI (from 27.3% to 21.0%) sites. DID estimate associated with this indicator was 5.6% (p=0.001) (Table 3). Temporal trends in the proportion of urban women that listened to family planning messages on the radio exhibited a significant decline in both ABRI (from 64.2% to 49.7%) and non-ABRI areas (from 66.9% to 39.8%). However, the decline was faster by about twice in non-ABRI areas and the associated DID estimate was 12.6% (p=0.006) (Table 3).

**Table 3:** Adjusted and unadjusted Difference-in-Difference (DID) estimates for source information about family planning, for all samples (total), rural and urban

	*Diff 2005= [ABRI 2005 - non-ABRI 2005]* *Diff 2016= [ABRI 2016 - nonABRI 2016]* *DID=Dif 2016- Diff 2005*	Total		Rural		Urban	

Indicators		Estimate (%)	p-value	Estimate (%)	P-value	Estimate (%)	P-value
Listened about family planning on the radio (past 12 months)	Unadjusted DID	5.6	<0.001	0.4	0.85	12.6	0.006
	Adjusted DID ^+^	3.2	0.055	0.2	0.919	11.0	0.012
Discussed family planning with a health worker in health facility (past 12 months)	Unadjusted DID	4.6	<0.001	3.6	0.013	8.7	0.009
	Adjusted DID^+^	4.5	0.001	3.5	0.015	8.1	0.012

+ DID with covariates, adjusted for women's age, education, religion, and parity.

From 2005 to 2016, the proportion of women who discussed family planning with a health worker in the past 12 months increased both in ABRI (from 6.3% to 18.8%) and non-ABRI areas (from 6.8% to 14.7%). The temporal increase was faster in ABRI than in non-ABRI areas, with an adjusted DID estimate of 4.5% (p=0.055). In ABRI areas, the proportion of women who reported discussing family planning with health worker increased at a faster rate both in urban and rural areas, with a DID estimate of 8.1% (P=0.012) and 3.6% (p=0.015), respectively (Table 3).

**Current use of family planning**: The mCPR increased both in ABRI (from 11.7% in 2005 to 41.8% in 2016) and in non-ABRI areas (from 13.5% in 2005 to 32.6% in 2016). The trend was much faster in ABRI areas, and the adjusted DID was 9.2% after accounting for the women's age, education, religion, and parity (p<0.001). Contraception use among rural women in ABRI and non-ABRI increased from 2005 to 2016 (ABRI 8.8% to 38.4%; non-ABRI 11% to 30.8%). The ABRI areas appeared to have a faster temporal trend in mCPR with an adjusted DID estimate of 9.4% at P<0.001. Injectable use trend was faster in ABRI areas than in the non-ABRI, as seen with a DID value of 6.3% (p<0.001). After adjusting for women's age, education, religion, and parity, the DID remained high (6.2%) and statistically significant (p<0.001) (Table 4).

**Table 4:** Adjusted and unadjusted Difference-in-Difference estimates for modern contraceptive prevalence rate, current use of LARC, Implants, and Injectables, for all samples, rural and “”prevalence rate (mCPR), by women's age

	mCPR by age					

	15-19 Yrs		20-24Yrs		25-49Yrs	
mCPR	Estimated (%)	P-value	Estimated (%)	P-value	Estimated (%)	P-value
Unadjusted DID	22.4	0.007	10.7	0.035	10.0	<0.001
Adjusted DID	16.7	0.037	9.2	0.052	8.4	<0.001

+ *DID with covariates, adjusted for education, religion, and parity*.

**Informed choice**: The proportion of current users that reported being told about method side effects exhibited a slight increase both in ABRI (from 31.4% to 39%) and in non-ABRI areas (from 32.4% to 40%). Similarly, the proportion that reported being informed about what to do if experiencing side effects increased in both ABRI (from 23.2% to 31.6%) and non-ABRI (from 25.4% to 31.1%) areas from 2005 to 2016, with a DID estimate of 2.7% (p>0.05). Additionally, the proportion of women who reported receiving information about other methods when given the current method increased both in the ABRI (from 29.6% to 45.8%) and in the non-ABRI (from 34.2% to 51.6%) from 2005 to 2016. Temporal trends did not differ significantly between the two areas with a DID value of 1.1% (p>0.05).

## Discussion

The findings from this secondary analysis of the EDHS indicate that the ABRI project contributed significantly to several family planning indicators in Ethiopia between 2005 and 2016. In particular, the analysis unearthed that overall contraceptive prevalence rate, use of injectables, women's knowledge of LARC methods, and their exposure to family planning information/messages from health workers all significantly improved in the ABRI intervention areas beyond what occurred in the non-ABRI area. These findings add to the literature on effective interventions that increase modern contraception knowledge and uptake and provide evidence for the ABRI project's success. This is a particularly important addition to the literature given the dearth of difference-in-difference analyses of family planning interventions in Ethiopia.

In the last two decades, Ethiopia has recorded dramatic success in increasing the utilization of family planning services and, as a result, the country is considered among the family planning success stories in sub-Saharan Africa (11). The significant increase in mCPR uptake in the ABRI intervention areas compared to the non-ABRI intervention areas (DID: 11%) indicates the important role that ABRI played in supporting this progress. Additionally, although the DID analysis found improvements among all age groups, the relative increasing trend was by far the highest among adolescents. Adolescent girls in Ethiopia face unique challenges in accessing family planning. Evidence suggests that social norms and stigma are some of the biggest barriers to adolescents accessing family planning (12–14). Furthermore, receiving youth-friendly services (13,15), and geographic proximity to a health center for rural adolescents (16) are associated with higher uptake of modern contraception among youth in Ethiopia. The work of ABRI in capacity building and support of health extension workers may have improved the youth-friendly care received and reduced proximity barriers that adolescent and youth age groups face when accessing contraception, which may explain the significant increase in ABRI areas. Furthermore, improved uptake of contraception through provision by community health workers is consistent with another study conducted in Ethiopia (17).

In addition to mCPR uptake, our findings highlight the success of the ABRI project in increasing the knowledge of LARCs and exposure to family planning information. It is claimed that knowledge of LARC is low in resource-limited settings (18). Socio-demographic characteristics of the women, such as education and residency, can influence the prevalence of contraception knowledge (19). In this study, the change persisted irrespective of women's age, education, religion, and parity.

ABRI used tailored approaches including targeted promotion and increased counseling and education. These approaches might increase access to LARC information for women with diverse socioeconomic demographics. Furthermore, evidence (20–22) reveals that barriers, such as structural (lack of trained personnel), the personal characteristics of clients (lack of knowledge), and health facilities readiness affect information access to LARCs-Implant and IUD. Our findings of remarkable change in Implant and IUD knowledge are likely attributed to the ABRI program strengthening and supporting health facilities to overcome barriers to inadequate information about contraceptive choices, and lack of trained personnel.

Although evidence on the efficacy of radio programs on the knowledge and uptake in Ethiopia and SSA is limited, evidence from sub-Saharan Africa suggests that exposure to family planning messages on the radio is associated with increased modern contraception uptake (23) and knowledge (24). This finding suggests that the ABRI project's radio program played an integral role in raising family planning awareness and uptake in ABRI initiative areas. Similarly, the significant technical support provided to primary health care units and the training of health care providers may have helped health workers reach more clients with family planning information. Discussing family planning with a health worker is associated with higher contraception uptake in Ethiopia (25), again pointing to the success of ABRI in increasing knowledge, and ultimately uptake, of modern contraception.

The limitations of the DID approach deserve to be mentioned. The Difference-in-Difference approach is generally less robust than analysis based on randomized selection. Bias in the estimation may appear if any exogenous factors are present that could influence the comparison of the differences in trends between the two groups. Difference-in-Difference methodology thus assumes that such factors are not present. In our analysis, however, we cannot ascertain the presence or absence of other competing intervention programs that could influence family planning outcomes in the ABRI intervention area. It is also possible that the ABRI intervention can have a spillover effect on the non-ABRI area, which could bias the comparison of the two groups towards the null. Additionally, because the ABRI project was multifaceted, it may be difficult to determine which specific aspects of the intervention contributed the greatest impact on various outcomes over time. This limitation restricts the ability to make specific programmatic recommendations for future initiatives in the area. Furthermore, because this analysis used EDHS data from 2005 and 2016, the study is subject to the limitations of these datasets, which may include reporting and recall bias (26).

In tandem with the Government of Ethiopia and other partners' programs, EngenderHealth has contributed its part to the recorded positive and significant changes in several family planning outcomes in the ABRI areas. The approaches that the ABRI project used to provide support at all levels, with a focus on strengthening woreda (district) health offices and health facilities through capacity building, were important in reaching women with the greatest need for contraception and were found to effectively contribute to improvements in family planning outcomes over time.

## References

[1] Cleland J, Bernstein S, Ezeh A (2006). Family planning: the unfinished agenda. The Lancet.

[2] United Nation (2016). Trends in Contraceptive Use Worldwide 2015.

[3] (2023). Ethiopia Population (2023) - Worldometer.

[4] (2006). National Reproductive Health Strategy 2006 - 2015.

[5] Central Statistical Agency (CSA) [Ethiopia] and ICF (2016). Ethiopia - Demographic and Health Survey 2016.

[6] Yalew SA, Zeleke BM, Teferra AS (2015). Demand for long acting contraceptive methods and associated factors among family planning service users, Northwest Ethiopia: a health facility based cross sectional study. BMC Research Notes.

[7] Fredriksson A, Oliveira GM de (2019). Impact evaluation using Difference-in-Differences. RAUSP Management Journal.

[8] The DHS Program - Demographic and Health Survey (DHS) (2023). https://dhsprogram.com/methodology/Survey-Types/DHS.cfm.

[9] Contraceptive prevalence rate (CPR) – DataForImpactProject (2023). https://www.data4impactproject.org/prh/family-planning/fp/contraceptive-prevalence-rate-cpr/.

[10] Mekonnen Y, Sahleyesus D (2019). Stalling of Contraceptive Prevalence Rate and Method mix trend in Addis Ababa, Ethiopia.

[11] USAID (2023). Three Successful Sub-Saharan Africa Family Planning Programs: Lessons for Meeting the MDGs.

[12] Dingeta T, Oljira L, Worku A (2021). Low contraceptive utilization among young married women is associated with perceived social norms and belief in contraceptive myths in rural Ethiopia. PLOS ONE.

[13] Jain A, Ismail H, Tobey E (2019). Stigma as a barrier to family planning use among married youth in Ethiopia. J Biosoc Sci.

[14] Ketema H, Erulkar A (2018). Married Adolescents and Family Planning in Rural Ethiopia: Understanding Barriers and Opportunities. Afr J Reprod Health.

[15] Teshome L, Belayihun B, Zerihun H (2021). Modern contraceptives use and associated factors among adolescents and youth in Ethiopia. Ethiopian Journal of Health Development.

[16] Habte A, Dessu S, Bogale B (2022). Disparities in sexual and reproductive health services utilization among urban and rural adolescents in southern Ethiopia, 2020: a comparative cross-sectional study. BMC Public Health.

[17] Asnake M, Henry EG, Tilahun Y (2013). Addressing unmet need for long-acting family planning in Ethiopia: uptake of single-rod progestogen contraceptive implants (Implanon) and characteristics of users. Int J Gynaecol Obstet.

[18] Tibaijuka L, Odongo R, Welikhe E (2017). Factors influencing use of long-acting versus short-acting contraceptive methods among reproductive-age women in a resource-limited setting. BMC Women's Health.

[19] Alemayehu M, Belachew T, Tilahun T (2012). Factors associated with utilization of long acting and permanent contraceptive methods among married women of reproductive age in Mekelle town, Tigray region, north Ethiopia. BMC Pregnancy Childbirth.

[20] Anguzu R, Tweheyo R, Sekandi JN (2014). Knowledge and attitudes towards use of long acting reversible contraceptives among women of reproductive age in Lubaga division, Kampala district, Uganda. BMC Research Notes.

[21] Umoh AV, Abah MG (2011). Contraception awareness and practice among antenatal attendees in Uyo, Nigeria. Pan Afr Med J.

[22] Family Health International (2007). Addressing Unmet Need for Family Planning in Africa: Long-Acting and Permanent Methods.

[23] Sserwanja Q, Turimumahoro P, Nuwabaine L (2022). Association between exposure to family planning messages on different mass media channels and the utilization of modern contraceptives among young women in Sierra Leone: insights from the 2019 Sierra Leone Demographic Health Survey. BMC Womens Health.

[24] Bekele D, Surur F, Nigatu B (2020). Knowledge and Attitude Towards Family Planning Among Women of Reproductive Age in Emerging Regions of Ethiopia. J Multidiscip Healthc.

[25] Mruts KB, Tessema GA, Dunne J (2022). Does family planning counselling during health service contact improve postpartum modern contraceptive uptake in Ethiopia? A nationwide cross-sectional study. BMJ Open.

[26] Boerma JT, Sommerfelt AE (1993). Demographic and health surveys (DHS): contributions and limitations. World Health Stat Q.

